# Evaluation of the performance and gas emissions of a tractor diesel engine using blended fuel diesel and biodiesel to determine the best loading stages

**DOI:** 10.1038/s41598-021-89287-0

**Published:** 2021-05-07

**Authors:** Haitham Emaish, Khamael M. Abualnaja, Essam E. Kandil, Nader R. Abdelsalam

**Affiliations:** 1 Department of Soils and Agricultural Chemistry, Biosystem Engineering, Faculty of Agriculture, (Saba Basha), Alexandria University, P.O. Box 21531, Alexandria, Egypt; 2 Department of Chemistry, College of Science, Taif University, P.O. Box 11099, Taif, 21944 Saudi Arabia; 3 Plant Production Department, Faculty of Agriculture, (Saba Basha), Alexandria University, P.O. Box 21531, Alexandria, Egypt; 4 Agricultural Botany Department, Faculty of Agriculture, (Saba Basha), Alexandria University, P.O. Box 21531, Alexandria, Egypt

**Keywords:** Biological techniques, Biophysics, Ecology, Biogeochemistry, Environmental sciences, Solid Earth sciences, Gastroenterology, Energy science and technology, Materials science

## Abstract

Fossil fuels are the main energy sources responsible for harmful emissions and global warming. Using biodiesel made from waste deep-frying oil as an alternative fuel source in diesel engines has drawn great attention. This biodiesel is produced using the transesterification process and blends with mineral diesel at Faculty of Agriculture Saba Basha, Alexandria University, Egypt. The turbocharged diesel engine of a Kubota M-90 tractor was tested. The objectives of this work are to test tractor as a source of power in the farm using waste deep-frying oil biodiesel to utilize waste frying oils (WFO) in clean energy production on the farm and determine the best engine loading stages to maximize engine efficiencies for different fuel blends and reduce the environmental impact of gas emissions from tractor diesel engines in the farms. The experiment design was factorial, with two factors, where the first was the engine load (0%, 25%, 50%, 75%, and 100%) and the second was fuel blend (0%, 5%, 20%, and 100% biodiesel), and the effects of loading stages and biodiesel percentage on engine performance indicators of engine speed, power take off torque, power take off power, brake power, brake mean effective pressure, brake thermal efficiency, brake specific fuel consumption, and gas emissions were studied. The experimental results indicated that engine load percentage and fuel blend percentage significantly affected all studied characters, and the best engine loading stages were between 25 and 75% to maximize engine efficiency and minimize the specific fuel consumption and gas emissions. Increasing the biodiesel percentage at all loading stages resulted decreasing in Engine brake power (BP), brake thermal efficiency, Power take-off (PTO) torque, and brake mean effective pressure and increases in brake specific fuel consumption. Increasing the engine load resulted in decreases in O_2_ emissions and increases in CO_2_, CO, NO, and SO_2_ emissions. Increasing the biodiesel percentage in the blended fuel samples resulted in increases in O_2_ and NO emissions and decreases in CO_2_, CO, and SO_2_ emissions. The use of biodiesel with diesel fuel reduces the environmental impact of gas emissions and decreases engine efficiency.

## Introduction

Fossil fuels are the main energy sources responsible for harmful emissions and global warming. Increases in energy consumption are generating a greater reliance on fossil fuels and are vastly increasing carbon dioxide emissions, leading to environmental pollution, especially in the transportation sector, where the highest consumption of liquid fuel is found^1^. Biodiesel is a clean fuel source for diesel engines, produced by a chemical reaction between a vegetable oil and methanol or ethanol alcohol in the presence of a catalyst^2^. Waste frying oil (WFO) are related for deep frying with high heat temperatures (150–190 °C), it is a spent vegetable oil that used for deep frying and no more viable for further consumption^3–5^. But cooking oils are large range of all oil types that used for common cooking^6^. Biodiesel is made from fresh vegetable oils, such as, palm oil, sunflower oil, soybean oil, and so on, which can result in increased food prices^7,8^. The cost of feedstock is about 80% of production costs of biodiesel, so vegetable oils are too high for biodiesel production^9,10^. To solve this problem, waste frying oil is the best choice for biodiesel production because it is low-cost feedstock and non-edible oils^11^. In addition, unsafe disposal of waste frying oil results in environmental pollution. Therefore, to solve this problem, WFO was used as feedstock to produce biodiesel^12,13^.

The main characteristics of biodiesel are quite like those of mineral diesel. Biodiesel is compatible with mineral diesel and can be mixed in with different ratios. The replacement of mineral diesel with vegetable oils in diesel engines has detected some problems, particularly due to the lower volatility of vegetable oils and their higher viscosity, density, and molecular weight. Hence, it has been reported that the “transesterification process is the best method and the most common technology to produce biodiesel”^14^. The quality and the yield of the produced biodiesel depend on the type of raw material, as well as the process parameters (oil and methanol molar ratio, the catalyst type and quantity, reaction time and temperature and finally blending speed^15^.

Biodiesel is an alternative energy source for internal combustion engines. Diesel engines can run on vegetable oil as fuel and produce equivalent power to that produced by mineral diesel. The biodiesel mass flow energy delivery increases with the higher density and viscosity of the vegetable oil^16^. Using biodiesel made from waste frying oil is an alternative fuel source in diesel engines which drawn great attention. A biodiesel sample was converted from waste cooking oils and tested in a diesel-powered bus on a dynamometer. The performance of biodiesel were similar to those of mineral diesel, with the exception of a significant reduction of emissions during acceleration with biodiesel^17^. The performance of diesel engines using blended fuel consisting of 20% by volume of waste cooking-oil biodiesel and 80% of mineral diesel indicated that engine thermal efficiency decreased, and specific fuel consumption increased with increased biodiesel blends relative to diesel fuel^2,18–20^.

The performance of tractors using different fuel blends of mineral diesel and biodiesel was conducted in stationary and non-stationary conditions indicate the power of engine and drawbar power which decreased with the use of biodiesel and different fuel blends of biodiesel, while resulted in increase the specific fuel consumption^21^.

The effects of using different blends of palm biodiesel on the performance of a diesel engine showed a reduction in carbon monoxide emissions and unburned hydrocarbons (HC) emissions of about 46 and 73%, respectively. Blended palm biodiesel (B_20_) causes a reduction in exhaust emissions relative to mineral diesel^22–24^. The performance and emissions of diesel engines have been studied with the use of mineral diesel and biodiesel produced from soybean oil. The specific gravity and viscosity of biodiesel were higher than those of diesel fuel at 40 °C. The heat of biodiesel combustion was 12% lower than that of mineral diesel. It was observed that smoke opacity and engine power were decreased by 71% and 4.8%, respectively, when the engine was operated with biodiesel compared to mineral diesel. However, the maximum engine torque was decreased by about 6 and 3.2% at 1700 and 1300 rpm, respectively. A gas emissions test showed that increasing the biodiesel percentage resulted in decreases in HC, carbon monoxide (CO), and oxides of nitrogen (NOx) by about 54, 46, and 14.7%, respectively, which resulted in increasing CO_2_ by about 0.5%^25^. This study was conducted to study the effects of blending waste frying oil (WFO) biodiesel with diesel fuel on the performance of a diesel engine and determine the best engine loading stages to maximize engine efficiencies for different fuel blends and reduce the environmental impact of gas emissions for diesel engines.

## Materials and methods

### Laboratory preparation of biodiesel

A waste frying oil (WFO) biodiesel sample was prepared and blended with mineral diesel in a laboratory at the Soil and Agricultural Chemistry Department, Faculty of Agriculture Saba Basha, Alexandria University, Egypt. The WFO was converted to biodiesel using transesterification. The amounts of methanol alcohol and catalyst (NaOH) were determined using the titration process to improve the reaction rate and yield^26^.The optimum biodiesel transesterification process parameters are (A) Removing impurities from the WFO using filter paper; (B) Heating oil to 100 °C to evaporate the water; (C) Cooling the oil to 60 °C to begin the reaction; (D) Molar ratio of 6:1 (6 mol of alcohol:1 mol of waste frying oil); (E) The catalyst also is commonly considered 1% of the oil weight unless is not determined from titration; (F) Mixing methanol and sodium hydroxide with the oil for 1 h and leaving them in a separator funnel for 1 h to separate the glycerol from the methyl esters; (G) Heating distilled water to 60 °C and slowly shaking it by hand with methyl esters before leaving it in a separator funnel for 15 min to separate suspended impurities and washing water from the methyl esters; (H) Washing the methyl esters with distilled water until the water was pure at pH values between 6 and 7 and (I) Heating the biodiesel to 100 °C to free it of water According to Ma and Hanna (1999) and Verma and Sharma (2016). Biodiesel properties are pour point − 7 °C according to the method ASTMD-97; Ash content wt% (Nil) according to ASTMD-482; Calorific value (Mj/kg) was 42.3 and Flash point 142 °C (ASTMD-93)^27^.

### Performance evaluation of Kubota M-90 tractor

Fuel blends were tested using a direct-injection turbocharger diesel engine for a Kubota M-90 tractor (Kabuto-cho, Chuo-ku, Tokyoas, Japan) a source of farm power for various agricultural operations in the field.

**Figure 1:**
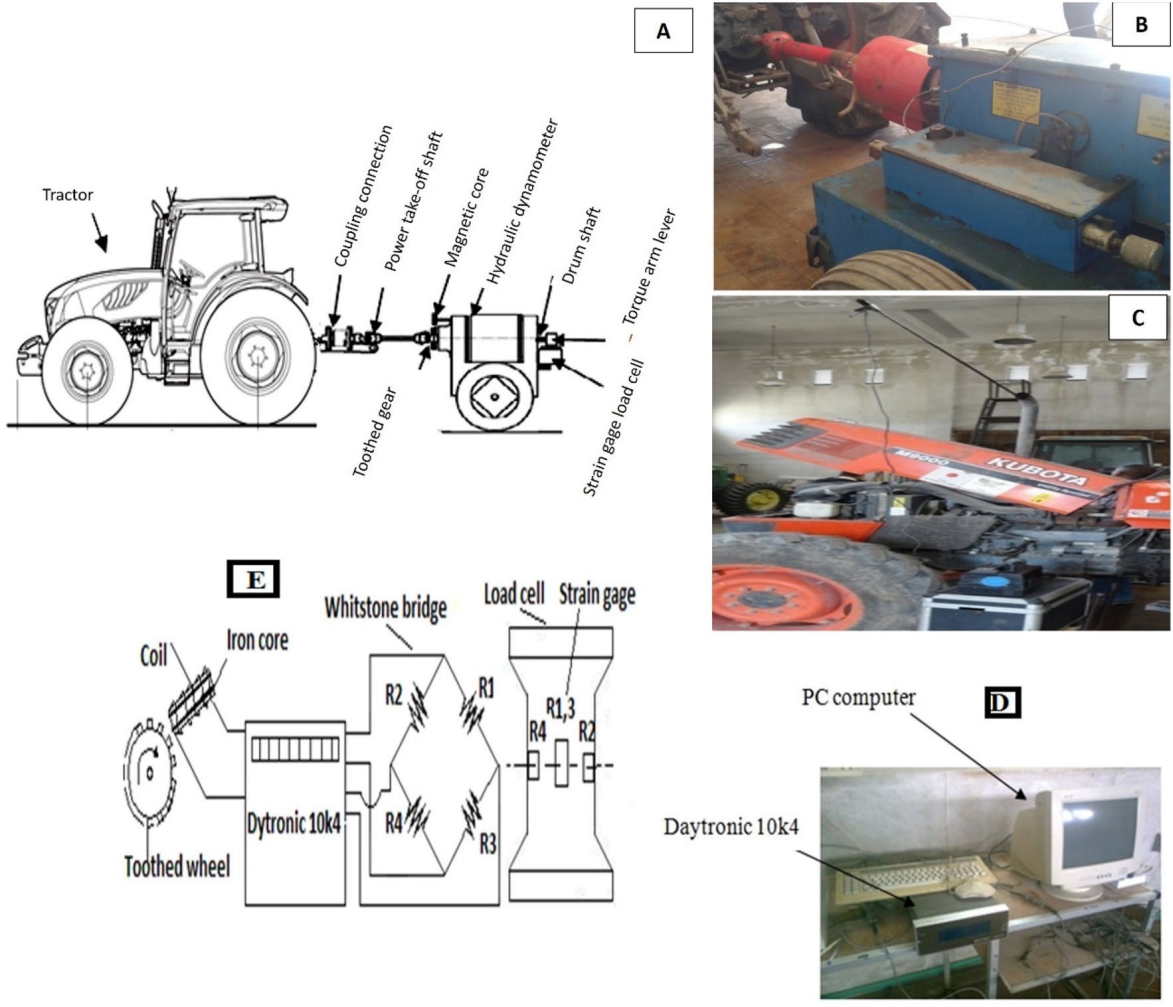
Tractor test equipment (**A**,**B**), connecting gas analyzer with the tractor (**C**), (**D-E**) A data acquisition system to save the measurements on the computer.

The performance of the direct-injection turbocharger diesel engine of the Kubota M-90 tractor (66.2 kW) was determined using a hydraulic brake stationary dynamometer (300 kW) at the Testing and Research Station for Tractors and Agricultural Machinery, of the Agricultural Research Center, Alexandria, Egypt as shown in Fig. 1a,b. The engine specifications are [Tractor model: M-90 4 WD, Engine type: Direct-Injection turbocharger, Engine model: V4702-TL water cooled, Number of cylinders: 4, Total displacement, cm^3^: 4665, Bore and stroke, mm: 109 × 125, Net power kW (hp): 66.2 (90), Power take-off (PTO) power kW (hp): 52.6 (71.5)/2400, and Max engine torque Nm/rpm: 335/1200)]. The engine was left to run for 1 h to warm up all the parts so that they reached the best working temperature before the test was begun. The tractor power take-off (PTO) shaft related to the brake dynamometer, and then the engine was run at full throttle for all tests and left running for 10 min to consume fuel residue after the fuel type was changed before each test. Five loading stages of 0, 25, 50, 75 and 100% were selected for every test. The engine started up at no loading stage with maximum speed; dynamometer sensors and Daytronic (model 10k4, https://www.daytronic.com/resources/software-manuals/) was used to record measured data by the pick-up frequency and torque sensor according to^28^, which engaged to the hydraulic dynamometer. The torque sensor comprises of a full whinstone bridge of strain gage for record the torque exerted on PTO shaft during loading test. The pick-up frequency sensor was used to recording the rpm of the PTO shaft, which comprises of a toothed wheel and coil around an iron core, the magnetic field of the magnetized cause is detected when the toothed wheel rotates and displayed as rpm. A data acquisition system was used to save the measurements on the computer, as shown in Fig. 1d,e, the load on the PTO shaft was increased by using a hydraulic dynamometer until a max load of 100% was reached, which led to a minimum speed. The dynamometer was equipped with torque and speed sensors to measure the torque and speed of the PTO shaft at each loading stage. The performance indicators of the turbocharged diesel engine were calculated using the measured torque, speed of PTO shaft, fuel consumption, and time for each test. The quality of gas emissions was measured with a portable gas analyzer (NOVA 7460), which consists of electrochemical sensors and infrared flue gas analyzers to measure the temperature and the gas elements content of (CO_2_, CO, NO, NO_x_, O_2_, and SO_2_). The gas analyzer was calibrated in the laboratory by using analyzed calibration gases and all values were Zero on air before connected to the tractor exhaust pipe during the test, as shown in Fig. 1c.

#### Gas analyzer specification

The Portable Analyzers (7460 Series) are presented in 6 versions for the simultaneous measurement of the gases normally found in the exhaust from internal combustion engines. HC, CO_2_, and CO are identified by a dual wavelength infrared sensor. The NOx, SO_2_ and O2 are identified by customer disposable electro chemical sensor. The optional low range CO channels and NO2 are identified by electrochemical sensor. The resolution is (0.1%); 1 PPM; accuracy and repeatability is 1% ( ±) of full scale for O2, CO, CO_2_, SO_2_ and HCs 2% ( ±) of full scale for NOx; available ranges is (0–5.00%/ 10.00% CO (standard range), (0–2000/5000/10,000 PPM CO (low range), (0–2000/5000 PPM NOx (as NO), (0–2000/5000/10,000/20,000 PPM HC's), (0–25.0% O2), (0–20.0% CO_2_) and (0–800 PPM NO2) and response time is 8–10 s**.**

### Experimental design

**Scheme 1:**
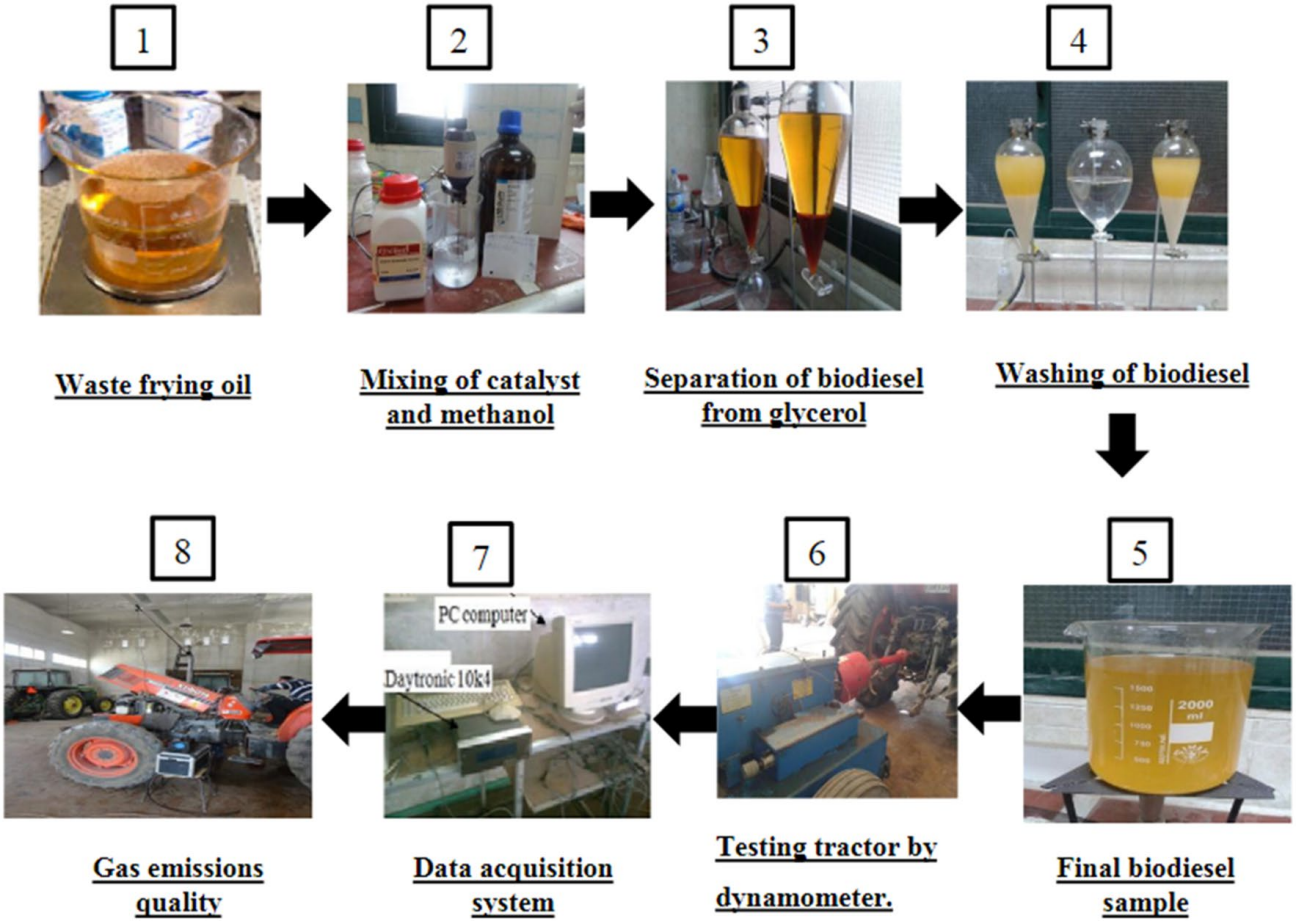
The experimental steps.

The experiments were conducted as factorial experiments in two factors: the first factor was the engine load of 0, 25, 50, 75, and 100%, and the second factor was fuel blends, percentage of B_0_ (100% diesel), B_5_ (5% biodiesel and 95% diesel), B_20_ (20% biodiesel and 80% diesel), and B_100_ (100% biodiesel). Each treatment was distributed across three replications (see Scheme 1).

### Performance indicators of diesel engine

#### PTO torque and power

The exerted power in the PTO shaft was calculated by measuring the torque and the rotational speed of the tractor PTO shaft using the following equation.

$$P = \frac{2 \cdot \pi \cdot n \cdot \tau }{{\text{c}}},$$

where P: power of PTO shaft (kW), n: rotational speed of PTO (rpm), τ: torque of PTO shaft (Nm) and c: constant equal to 60,000^29^.

#### Fuel consumption

The fuel consumption was determined by measuring the volume of consumed fuel and the time spent during each loading stage of the test, as given in the following equation:

$${\text{FC }} = \frac{{{\text{V * }}3600}}{{{\text{t * }}1000}},$$

where V: volume of consumed fuel (cm^3^), t: time of the test (s) and FC: fuel consumption rate (L/h)^29^.

#### Engine brake power

Engine brake power (BP) is the actual power of the engine, determined from the force exerted on a dynamometer connected to the PTO shaft. BP was calculated according to the technical data of the Kubota tractor as 142.5% of the power at PTO; the ratio between the engine and PTO shaft speeds (rpm) was (2205/540), or 4.0833:1^29^.

$${\text{BP}} = \frac{{\text{PTO power}}}{0.7},{\text{ kW}}$$

#### Brake thermal efficiency

Brake thermal efficiency (BTE) is the ratio of the BP to the amount of heat energy produced from burning 1 kg fuel in the engine, which can be calculated using the following equation.

$${\text{BTE}} = \frac{BP*3600}{{FC*\rho_{f} *HV}},$$

where BP: brake power (kW), $\rho_{f}$: fuel density (kg/L), FC: fuel consumption, (L/h) and HV: fuel heat value (kJ/kg)^29^.

#### Brake specific fuel consumption

Brake specific fuel consumption (BSFC) is the consumed fuel (FC) per kg to produce BP of 1 kW at 1 h, which can be calculated by dividing the fuel consumption (kg/h) by the corresponding BP (kW) in the same loading conditions, represented by different levels of engine speed, assessed with the following equation (according to^29^).

$${\text{BCFC}} = \frac{{{\text{FC}}}}{{{\text{BP}}}} \times \frac{{{\text{kg}}}}{{{\text{kWh}}}},$$

#### Brake mean effective pressure

Brake mean effective pressure (BMEP) is the average pressure inside a cylinder on the surface of the piston during movement from the top to the bottom of the cylinder at each power stroke, which can be calculated using the following equation.

$${\text{BMEP}} = \frac{BP*2*60}{{L*A*n*N}},$$

where BMEP: brake mean effective pressure, kPa, L: piston displacement, m, A: piston cross-sectional area, m^2^, N: engine rotation speed, rpm, N: number of engine cylinders, 2: constant for four-stroke engine and 60: constant for unit conversion, s,^29^.

### Statistical analysis

All collected data were subjected to analysis of variance using the technique of^30^. All statistical analyses were performed according to Duncan^31^.

### Compliance with ethical requirements

This article does not contain any studies with human or animal subjects.

## Results and discussion

### Performance evaluation of tractor engine

The performance of the direct-injection turbocharger diesel engine for the Kubota M-90 tractor was evaluated at different engine loads with the use of different biodiesel blends with mineral diesel to maximize the engine efficiencies of PTO torque, BP, BMEP, and BTE, while also minimizing specific fuel consumption, gas emissions, and, finally, fossil fuel consumption. The results in Table 1 showed a significant effect of engine load percentage and fuel blend percentage and their interaction on all the studied characters.

**Table 1:** Effects of engine load percentage and fuel blends percentage on power take-off speed, power take-off power, power take-off torque, engine speed, brake power, brake specific fuel consumption, brake thermal efficiency, fuel consumption, brake mean effective pressure, O_2_ percentage, CO_2_ percentage, CO, NO, and SO_2_.

Treatments	PTO speed (rpm)	PTO power (kW)	PTO torque (Nm)	Engine speed (rpm)	Brake power (kW)	Brake specific fuel consumption (BSFC)	Brake thermal efficiency (BTE)	Fuel cons (kg/h)	brake means effective pressure (kPa)	O_2_ (%)	CO_2_ (%)	CO (ppm)	NO (ppm)	SO_2_ (ppm)
**(A) Load (%)**														
0	679.4a	7.0d	98.6e	2774.1a	10.0 d	1.01b	8.5d	10.2e	92.9e	15.27a	4.20e	86.00c	272.5e	2.00e
25	628.3b	24.9b	378.6d	2565.5b	35.6 b	0.42d	20.7b	14.9c	356.7d	10.99b	7.37d	62.53d	450.8d	7.31d
50	509.6c	29.6a	555.5c	2080.6c	42.4a	0.40e	22.0a	16.7a	523.1c	7.95c	9.68c	86.25c	548.8b	11.00c
75	323.3d	21.3c	627.9a	1320.2d	30.4c	0.52c	16.8c	15.6b	591.5a	6.13d	11.11b	157.16b	566.5a	13.06b
100	69.2e	4.3e	597.4b	282.6e	6.2 e	1.88a	4.6e	11.6d	563.1b	5.53e	11.68a	275.25a	504.0c	13.50a
LSD _0.05_	4.9	0.2	3.3	12.1	0.3	0.01	0.1	0.1	4.7	0.03	0.06	0.83	2.2	0.06
**(B) Blends (%)**														
0	454.3a	18.9a	475.6a	1854.8a	26.9a	0.79d	16.7a	13.1d	448.2a	8.75d	9.03	159.50a	460.0d	14.75a
5	447.0b	18.0b	460.3b	1825.2b	25.7b	0.82c	15.0b	13.7c	433.6b	9.06c	8.90b	152.75b	463.5c	12.75b
20	435.6c	16.7c	442.1c	1778.6c	23.9c	0.87b	13.7c	13.9b	416.4c	9.22b	8.93b	138.50c	466.5b	10.00
100	430.9d	16.1d	428.5d	1759.8d	23.0d	0.90a	12.6d	14.4a	403.6d	9.67a	8.38c	83.00d	484.0a	0.00
LSD_0.05_	4.4	0.2	3.0	10.8	0.2	0.01	0.1	0.1	4.2	0.03	0.05	0.74	2.0	0.06
**Interaction**														
A × B	*	*	*	*	*	*	*	*	*	*	*	*	*	*

Means in column (s) followed by the same letter are not significant at the 0.05 level of probability.

*Significant difference at the 0.05 level of probability.

#### Engine speed

**Figure 2:**
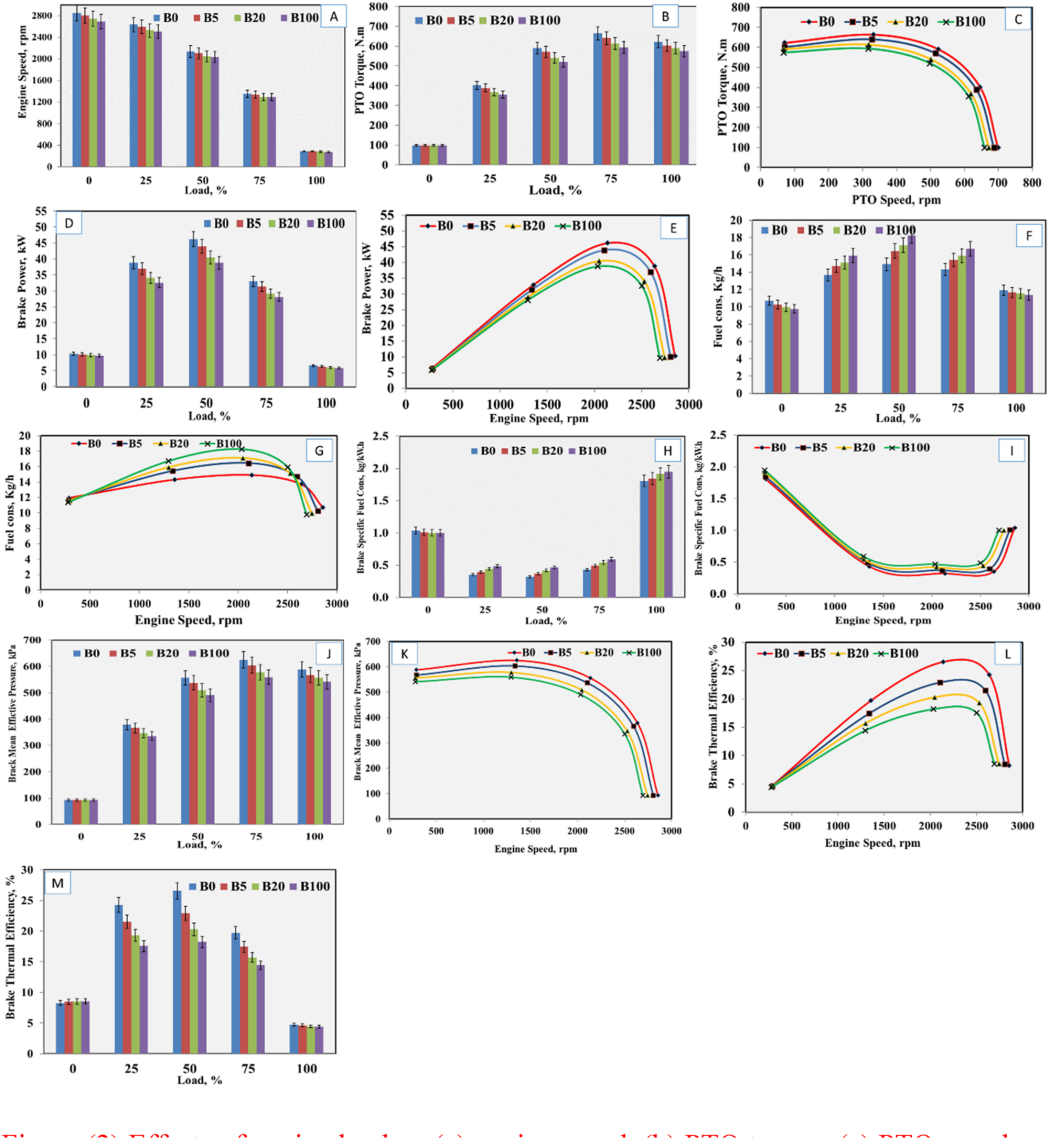
Effects of engine load on (**a**) engine speed, (**b**) PTO torque, (**c**) PTO speed on PTO torque, (**d**) engine load on brake power, (**e**) engine speed on brake power, (**f**) engine load on fuel consumption, (**g**) engine speed on fuel consumption, (**h**) engine load on (BSFC). (**i**) engine speed on (BSFC), (**j**) engine load on BMEP, (**k**) engine speed on BMEP, (**l**) engine speed on BTE and (**m**) engine load on BTE.

For the effects of engine load percentage on engine speed, the results in Table 1 indicated the relationship between engine load percentage and engine speed in rpm was inversely proportional. The maximum engine speed was recorded at a loading of 0%, and the lowest speed was at a loading of 100% (Table 1). Using 100% diesel fuel (B_0_) gave the highest engine speed among all treatments, and the lowest speed was recorded with 100% biodiesel fuel (B_100_). The significant interaction between engine load, percentage, and engine speed in rpm was inversely proportional, as shown in Fig. 2a. The maximum engine speed was 2854 rpm at the loading stage of 0% using 100% diesel fuel (B_0_), while the minimum speed was 276 rpm at the loading stage of 100% using 100% biodiesel fuel (B_100_), as shown in Table 2. At all loadings, stages with increased biodiesel percentages in the blended fuel samples resulted in decreased engine speed because the heating value of biodiesel is lower than that of mineral diesel^32–35^.

**Table 2:** Interaction effects between engine load percentage and fuel blends percentage on power take-off speed, power take-off power, power take-off torque, engine speed, brake power, brake specific fuel consumption, brake thermal efficiency, fuel consumption, brake mean effective pressure, O_2_ percentage, CO_2_ percentage, CO, NO, and SO_2_.

Engine load (%)	Biodiesel blends (%)	PTO speed (rpm)	PTO power (kW)	PTO torque (Nm)	Engine speed (rpm)	Brake power (kW)	Brake specific fuel consumption (BSFC)	Brake thermal efficiency (BTE)	Fuel cons (kg/h)	Brake means effective pressure (kPa)	O_2_ (%)	CO_2_ (%)	CO (ppm)	NO (ppm)	SO_2_ (ppm)
**Treatments**															
0	0	699.2	7.2	98.5	2854.8	10.3	1.04	8.3	10.7	92.8	15.30	4.20	99.00	281.0	4.00
	5	687.0	7.1	98.6	2805.0	10.1	1.01	8.5	10.3	92.9	15.20	4.18	91.00	274.0	3.00
	20	672.0	6.9	98.7	2743.8	9.9	1.00	8.5	9.9	92.9	15.28	4.20	85.00	269.0	1.00
	100	659.6	6.8	98.7	2692.9	9.7	1.00	8.5	9.8	93.0	15.30	4.20	69.00	266.0	0.00
25	0	646.0	27.2	401.9	2637.6	38.8	0.35	24.3	13.7	378.6	10.98	7.39	54.75	449.5	11.63
	5	635.5	25.9	389.2	2594.5	36.9	0.40	21.5	14.7	366.6	10.98	7.34	54.13	450.3	10.13
	20	619.3	23.8	367.7	2528.7	34.1	0.44	19.3	15.1	346.4	11.03	7.34	60.25	452.3	7.50
	100	612.5	22.8	355.7	2500.9	32.6	0.49	17.5	15.9	335.1	11.01	7.41	81.00	451.0	0.00
50	0	523.4	32.3	590.7	2137.2	46.2	0.32	26.6	14.9	556.1	7.70	9.80	85.00	539.0	17.00
	5	515.4	30.8	570.3	2104.4	43.9	0.37	2.9	16.4	537.3	7.90	9.70	85.00	545.0	15.00
	20	501.2	28.3	540.2	2046.4	40.5	0.42	20.3	17.1	508.9	8.00	9.70	87.00	551.0	12.00
	100	498.3	27.1	520.6	2034.4	38.8	0.47	18.2	18.2	490.2	8.20	9.50	88.00	560.0	0.00
75	0	332.0	23.1	663.9	1355.7	32.9	0.43	19.7	14.3	625.4	5.48	11.44	189.75	549.5	20.13
	5	327.2	21.9	640.9	1335.7	31.4	0.49	17.4	15.4	603.8	5.98	11.25	183.63	558.3	17.63
	20	317.2	20.4	613.3	1295.7	29.1	0.55	15.7	15.9	577.7	6.19	11.29	165.25	565.3	14.50
	100	316.8	19.7	593.3	1293.6	28.1	0.59	14.4	16.7	558.9	6.86	10.46	90.00	593.0	0.00
100	0	70.7	4.6	622.8	288.7	6.6	1.81	4.7	11.9	588.0	4.30	12.30	369.00	481.0	21.00
	5	70.1	4.4	602.3	286.2	6.3	1.85	4.6	11.7	567.4	5.20	12.00	350.00	490.0	18.00
	20	68.2	4.2	590.4	278.5	6.0	1.92	4.5	11.5	556.2	5.60	12.10	295.00	495.0	15.00
	100	67.8	4.1	573.9	276.9	5.8	1.95	4.4	11.3	540.9	7.00	10.30	87.00	550.0	0.00
LSD at 0.05		9.7	0.3	6.6	24.3	0.5	0.01	0.2	0.1	9.4	0.07	0.12	1.66	4.4	0.13

#### PTO torque

The results presented in Table 1 showed the significant effect of load percentage on PTO torque, where a loading stage of 75% achieved the highest PTO torque among all loading stage percentages, and the lowest value for PTO torque was obtained with a loading stage of 0%. Regarding the effects of fuel blend percentage on PTO torque, the results in Table 1 indicated that the fuel blends significantly affected PTO torque, and the highest value of this trait was achieved with B_0_ blend (100% diesel fuel) in comparison to the other blend percentages, while the lowest PTO torque was given with 100% biodiesel. The relationship between the torque of PTO shaft, Nm, and PTO load in percentage, and speed in rpm are shown in Fig. 2b,c, respectively. Increased PTO load resulted in decreased PTO speed and increased PTO torque until maximum torque values were reached for all blended fuel samples at a loading stage of 75% and a speed between 316 and 332 rpm, and then the torque decreased incrementally until the maximum loading stage was reached at a minimum PTO speed. Table 2 presents the results of the interactions between engine load percentage and fuel blend percentage, indicating that the maximum PTO torque was 663 Nm at a loading stage of 75% and PTO speed of 332 rpm, using 100% diesel fuel (B_0_), and the minimum PTO torque was 98.51 Nm at loading stage of 0% and PTO speed of 699.19, rpm using 100% diesel fuel (B_0_). At all loading stages, increasing biodiesel percentage in the blended fuel samples resulted in decreased PTO torque, which, due to the heating value of biodiesel, was lower than that of diesel fuel^34–36^. The values for PTO torque were close at different biodiesel percentages at the loading stage 0%, but engine performance cannot be judged at the no load stage with minimum torque, so the PTO load should be increased to see the difference between fuel types.

#### Engine brake power

Data in Table 1 showed that engine load percentage significantly affected BP, kW, such that engine load of 50% achieved the highest BP, and the lowest value for BP was obtained with 100% load. The results given in Table 1 show that fuel blend percentage was significantly affected BP, whereas the highest value for this trait was achieved with the 0% blend (100% diesel fuel) in comparison to the other blend percentage, while the lowest BP was given with 100% biodiesel. The interactions among BP, engine load, and engine speed were significant and were as presented in Fig. 2d, e. Moreover, increased engine load resulted in decreased engine speed and increasing BP until the highest value was reached at the loading stage of (50%) at engine speeds of 2034–2137 rpm for all fuel types shown in Table 2, which was due to the increased mass of burning fuel. The BP decreased until engine stop at a maximum loading stage of 100%, which was due to the effects of higher frictional force at the maximum loading stage^33–35,37^. The maximum BP was 46.2 kW at a loading stage of 50% and a speed of 2137 rpm at 100% diesel fuel (B_0_), while the minimum BP was 5.82 kW at a maximum loading stage of 100% and a speed of 276 rpm using 100% biodiesel (B_100_). At all loading stages, increased biodiesel percentages resulted in decreased BP because the calorific value of biodiesel was lower than that of diesel, as noted.

#### Fuel consumption

Data in Table 1 showed that engine load percentage affected significantly fuel consumption; 50% load achieved the highest fuel consumption, and the lowest value for fuel consumption was obtained for 0% load. The results in Table 1 showed that fuel blends percentage significantly affected fuel consumption, and the highest value of fuel consumption was recorded with the B_100_ blend (100% biodiesel fuel), and the lowest was given with the B_0_ blend of 100% diesel fuel. The significant interaction between fuel consumption, kg/h (Kilogram per hour) and each of engine load and speed are shown in Fig. 2f,g and the interaction between engine load percentage and fuel blend percentage are shown in Table 2, such that increased engine load resulted in decreased engine speed and increased fuel consumption until reaching the maximum value at a loading stage of 50% at maximum BP, which was because of the increased mass of burning fuel at this stage, and then the fuel consumption decreasing until reaching maximum loading^33–35^. The maximum fuel consumption was 18.24 kg/h at an engine speed of 2034.35 rpm using 100% biodiesel B_100_ at a loading stage of 50%. The minimum fuel consumption was 9.76 kg/h at an engine speed of 2692.9 rpm using 100% biodiesel B_100_ at a no-load stage. At loading stages between 0 and 100%, increasing biodiesel percentage resulted in increased fuel consumption, which is because the density of biodiesel was higher than that of diesel fuel.

#### Brake specific fuel consumption

The results in Table 1 indicated that engine load percentage significantly affected BSFC, such that the highest BSFC was achieved with an engine load of 100%, while the lowest value was obtained with an engine load of 50%. Other results shown in Table 1 indicated that increased biodiesel percentage in fuel blends produced significantly increased BSFC, and the maximum value of BSFC was given with B_100_ (100% biodiesel fuel); the lowest was seen with B_0_ percentage (100% diesel fuel). The relationship of interaction between (BSFC), (Kilogram per kilowatt hour) kg/kWh, engine load, and engine speed are shown in Fig. 2h,i, indicating that increased engine load resulted in decreased engine speed and BSFC until the minimum value was reached at a loading stage of 50% at maximum BP and fuel consumption. Then, the BSFC increased until it reached maximum value at a maximum loading stage of 100%, which was due to the highest frictional force and the lowest BP occurring at this loading stage^33–35^. The maximum (BSFC) was 1.95 kg/kWh at a loading stage of 100% and an engine speed of 276 rpm using 100% biodiesel fuel (B_100_); the minimum BSFC was 0.32 kg/kWh at an engine speed of 2137 rpm and a loading stage of 50% using 100% diesel fuel (B_0_), as shown in Table 2. At all loading stages, increased biodiesel percentages resulted in increased BSFC, except at the no loading stage. This is because the fuel consumption for biodiesel was higher than that for mineral diesel. Additionally, the calorific value of biodiesel was lower than that for diesel fuel, and the viscosity of the biodiesel was higher than that for mineral diesel, which leads to unfavorable pumping and spray characteristics^36,38^.

#### Brake mean effective pressure

The results in Table 1 indicated a significant effect of engine load percentage on BMEP, such that the highest BMEP was given by an engine load of 75%, and the other side the lowest value for BMEP was obtained for an engine load of 0%. The results given in the same table indicated that increased biodiesel percentage in fuel blends significantly decreased BMEP. The maximum value of BMEP was given with the 0 blend (100% diesel fuel), and the lowest BMEP was given with 100% biodiesel fuel. The interaction between BMEP, kPa, engine load, and engine speed are shown in Fig. 2j,k. The data in Table 2 show the interaction between engine load percentage and fuel blend percentage. It can be clearly seen that increased engine load resulted in decreased engine speed and increased BMEP until the maximum value was reached at a loading stage of 75% at engine speeds between 1293 and 1355 rpm. The BMEP decreased with slight values until reaching the maximum loading stage at minimum engine speeds between 276 and 288 rpm. The maximum BMEP was 625 kPa at an engine speed of 1355 rpm, using 100% diesel fuel (B_0_) at a loading stage of 75%. The minimum BMEP was 92 kPa at an engine speed of 2692 rpm, using 100% biodiesel (B_100_) at no loading stage. At all loading stages, increased biodiesel percentage resulted in decreased BMEP, except that there was no loading stage at which the BSFC did not change with different biodiesel percentages. This is because the effect of increased engine speed resulted in a decreased time remaining for combustion and resulted in an insufficient motion of air in the cylinder. Both effects decreased the combustion efficiency and the BMEP values, as shown in Fig. 2j according to^33–35,39^.

#### Brake thermal efficiency

The results shown in Table 1 cleared that engine load percentage significantly affected BTE; the highest BTE was recorded with a 50% load, and the lowest one was given with a 0% load percentage. Table 1 also indicated that fuel blend percentage significantly affected BTE, and the maximum value for BTE was given with 0 blend (100% diesel fuel). The lowest value was obtained with 100% biodiesel (B_100_). The relationship between BTE and engine load and engine speed are shown in Figs. 2l,m. Increased engine load caused decreased engine speed and increased BTE until the maximum value was reached at a loading stage of 50%; BTE decreased until a minimum value was reached at a maximum loading stage of 100% and minimum engine speeds between 276 and 288 rpm. The maximum BTE was 26% at a speed of 2137.17 rpm using 100% diesel fuel (B_0_) at loading stage of 50%. The minimum BTE was 4.4% at speed of 276 rpm, using 100% biodiesel (B_100_) at the maximum loading stage of 100%, as shown in Table 2. For all loadings stages increased biodiesel percentage resulted in decreased BTE, except at the no loading and maximum loading stages, where the BTE did not change with different biodiesel percentages. This is because the density of waste frying oil biodiesel was higher than that of diesel fuel, while its calorific value and volatility was lower, such that the combustion characteristics of biodiesel were lower than those of diesel fuel^34–36,40^.

### Gas emissions quality

**Figure 3:**
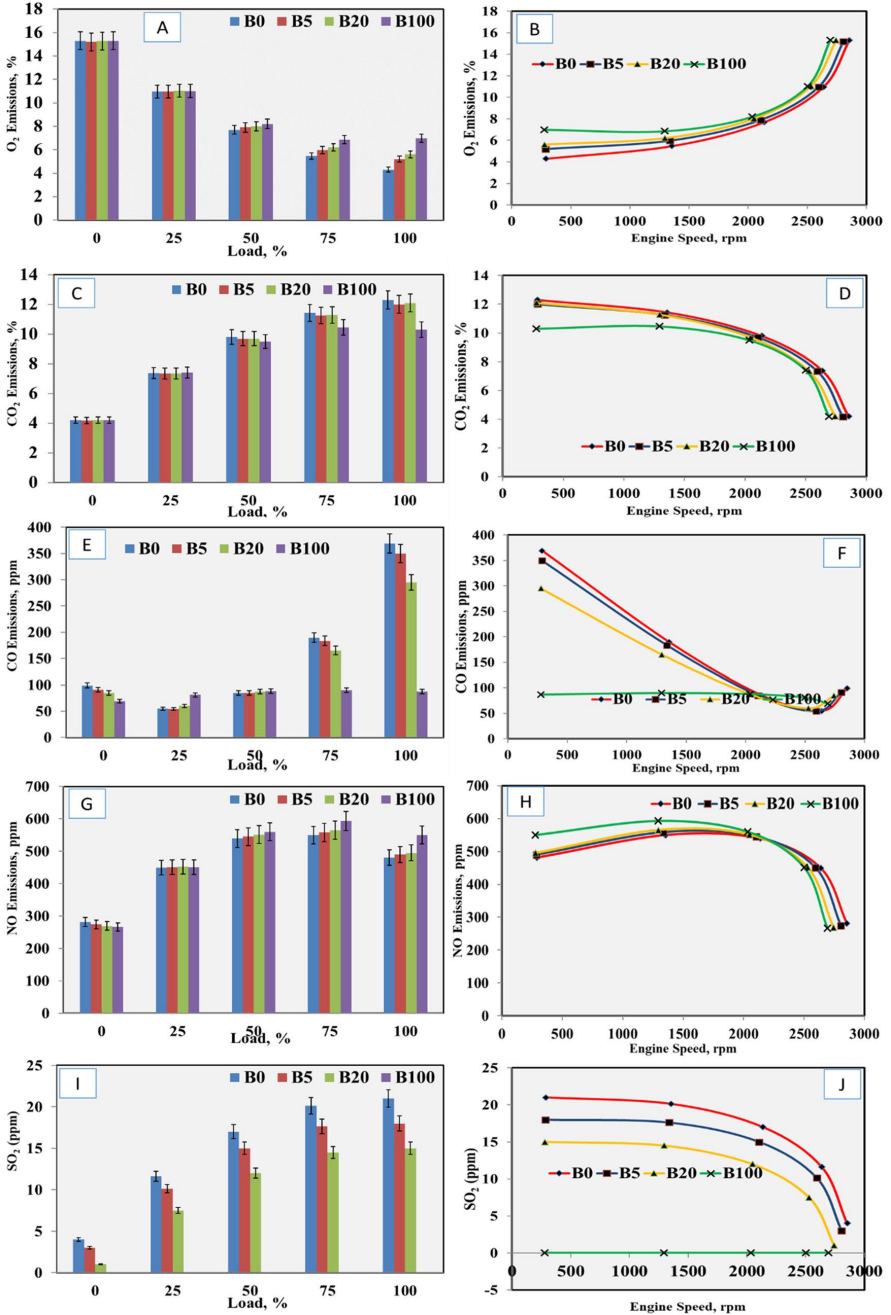
Effects of engine load on (**a**) engine load on O_2_ emissions, (**b**) engine speed on O_2_ emissions, (**c**) engine load on CO_2_ emissions, (**d**) engine speed on CO_2_ emissions, (**e**) engine load on CO emissions, (**f**) engine speed on CO emissions, (**g**) engine load on NO emissions, (**h**) engine speed on NO emissions, (**i**) engine load on SO_2_ emissions and (**j**) engine speed on SO_2_ emissions.

The results in Table 1 showed that an engine load of 0% significantly increased O_2_ emissions, and fuel blends of 100% biodiesel also increased O_2_ emissions relative to the other treatments. The relationships between O_2_ emissions, biodiesel percentage, engine load, and engine speed are shown in Fig. 3a,b. Increased engine load resulted in decreased O_2_ emissions because of the increased engine consumption of O_2_ to optimize fuel combustion, while increased engine speed resulted in increased O_2_ emissions. The maximum O_2_ emissions were 15.3% at the minimum loading stage for all fuel blends, while the minimum O_2_ emissions were 4.3% at maximum loading stage for 100% diesel fuel, as presented in Table 2. At all loading stages, increased biodiesel percentage in the blended fuel samples resulted in increased O_2_ emissions, except at the no loading stage, where the oxygen content in the biodiesel was about 10 to 12% higher than that of diesel fuel^34,35,41,42^.

The results in Table 1 showed that engine loading of 100% significantly increased CO_2_ and CO emissions, and the fuel blend of 100% diesel fuel (B_0_) increased CO_2_ and CO emissions relative to other treatments. The relationship between CO_2_ emissions, engine load, and engine speed are presented in Fig. 3c,d and Table 2. Increased engine load resulted in increased CO_2_ emissions until a maximum loading stage of 100% was reached, while increased engine speed resulted in decreased CO_2_ emissions. The maximum value for CO_2_ emissions was 12.3% at the maximum loading stage using 100% diesel (B_0_), and the minimum CO_2_ emissions was 4.2% at the no loading stage for all fuel blends. At loading stages of 50, 75, and 100%, increased biodiesel percentage in the blended fuel samples resulted in decreased CO_2_ emissions, which due to the oxygen content in the biodiesel was about 10–12%. A higher oxygen content contributes to increasing ignition quality and decrease CO_2_ emissions^35,36,41^.

The relationship between CO emissions, biodiesel percentage, engine load, and engine speed are shown in Fig. 3e,f. For all tested fuel samples, increased engine load resulted in a greater increase in CO emissions, until a maximum load was reached except at 100% biodiesel (B_100_), which increased slightly. Increased engine speed resulted in a sharp decrease in CO emissions until the maximum speed was reached, except at B_100_, which decreased slightly^43–45^. The maximum CO emissions value was 369 ppm at the maximum loading stage using 100% diesel fuel (B_0_), while the minimum CO emissions was 69 ppm at the minimum loading stage using 100% biodiesel fuel (B_100_). At all loading stages, increased biodiesel percentage resulted in decreased CO emissions except that at loading stages of 25% and 50%, for which the values of CO emissions were close. This was because high oxygen content in biodiesel increases ignition quality and decreases CO emissions, so increased biodiesel percentages reduce environmental pollution^36,43–46^. The results in Table 2 showed that an engine load of 75% significantly increased NO emissions, and 100% biodiesel fuel (B_100_) increased NO emissions relative to the other treatments.

The relationship between NO emissions, engine load, and engine speed are shown in Fig. 3g,h. Increased engine load resulted in decreased engine speed and increased NO emissions until the maximum value was reached at a loading stage of 75%. NO emissions decreased until reach a loading stage of 100% was reached with a minimum engine speed. The maximum NO emissions were 593 ppm at a loading stage of 75% using 100% biodiesel fuel (B_100_), while the minimum NO emissions were 266 ppm at the minimum loading stage using (B_100_) as showed in Table 1. At all loading stages, increased biodiesel percentages in the blended fuel samples resulted in increased NO emissions, except at the no loading stage, which was due to the increased burned fuel, which resulted in increased cylinder temperature. This was responsible for thermal NOx formation. Higher flame and cylinder temperatures with high oxygen content in the biodiesel led to higher NOx^36,43–46^. Table 2 shows that the engine load of 100% significantly increased SO_2_ emissions, and 100% diesel fuel increased SO_2_ emissions, relative to the other treatments.

The relationship between SO_2_ emissions, diesel percentage, engine load, and engine speed are shown in Fig. 3I,j and Table 1. Increased engine load resulted in increased SO_2_ emissions, and increased engine speed resulted in decreased SO_2_ emissions. There were no SO_2_ emissions by using 100% biodiesel (B_100_). The maximum SO_2_ emissions was 21 ppm at maximum loading stage using 100% diesel (B_0_). At all loading stages increasing biodiesel percentage in the blended fuel resulted in decreasing SO_2_ emissions^43–46^.

## Conclusions

In this study, a turbocharged diesel engine for a Kubota M-90 tractor was tested using different blends of WFO biodiesel with mineral diesel. The conclusions can be summarized as (1) the best engine loading stages in terms of engine efficiencies of PTO torque, PTO power, BP, BMEP, and BTE were between 25 and 75%, as well as for minimizing specific fuel consumption, gas emissions, and fuel consumption; (2) the engine loading stages of 0 and 100% did not clarify the difference between fuel blends, and the engine performance was low at these two stages, so using the tractor with a load lower than 25% or higher than 75% is inefficient; (3) using the tractor at a maximum loading stage of 100% resulted in an increase in engine vibration and noise, so it is not recommended to use this loading stage for any fuel blend. The best engine performance parameters were determined using diesel fuel, and while increasing the biodiesel percentage in the blended fuel samples resulted in worsening the performance parameters; (4) The maximum BP and BTE were 46.2 kW and 26%, respectively, at a loading stage of 50% and speed of 2137 rpm, using 100% diesel fuel (B_0_); (5) the best loading stages for the economical operation of the tractor were at the lowest BSFC at loading stages from 25 to 75% for all fuel blends and the maximum BSFC was 1.95 kg/kWh at loading stage of 100% and engine speed of 276 rpm using 100% biodiesel fuel (B_100_). the minimum (BSFC) was 0.32 kg/kWh at an engine speed of 2137 rpm, at a loading stage of 50% and using 100% diesel fuel (B_0_); (6) the maximum PTO torque and BMEP were 663 N/m at a PTO speed of 332 and 625 kPa and an engine speed of 1355 rpm at loading stage of 75% using 100% diesel fuel (B_0_), while the minimum PTO torque and BMEP were 98.51 N/m at PTO speed of 699 rpm and 92.78 kPa at an engine speed of 2854.7 rpm, respectively, at no loading stage using 100% diesel fuel (B_0_). At all loading stages, increased biodiesel percentage resulted in decreased PTO torque and BMEP; (7) increased engine load resulted in decreased O2 emissions and increased CO_2_, CO, NO, and SO_2_ emissions. The increased biodiesel percentage in the blended fuel samples resulted in increasing O2 and NO emissions, which resulted in decreased CO_2_, CO, and SO_2_ emissions and (8) the performance of the diesel engine using diesel fuel was higher than using WFO biodiesel. The environmental impact assessment for biodiesel was better than for diesel, which was because the CO_2_, CO, and SO_2_ emissions for biodiesel were lower than diesel.
